# Current opportunities to catalyze research in nutrition and cancer prevention – an interdisciplinary perspective

**DOI:** 10.1186/s12916-019-1383-9

**Published:** 2019-07-30

**Authors:** The Cancer Research UK - Ludwig Cancer Research Nutrition and Cancer Prevention Collaborative Group

**Affiliations:** 1 Cancer Research UK, Angel Building, 407 St John Street, London, EC1V 4AD UK; 2 Ludwig Cancer Research, 666 Third Avenue, New York, NY USA

**Keywords:** Cancer prevention, Diet, Nutrition, Physical activity, Obesity, Metabolism, Interdisciplinary, Epidemiology, Epigenetics, Developmental origins, Public health

## Abstract

Cancer Research UK and Ludwig Cancer Research convened an inaugural international Cancer Prevention and Nutrition Conference in London on December 3–4, 2018. Much of the discussion focused on the need for systematic, interdisciplinary approaches to better understand the relationships of nutrition, exercise, obesity and metabolic dysfunction with cancer development. Scientists at the meeting underscored the importance of studying the temporal natural history of exposures that may cumulatively impact cancer risk later in life.

A robust dialogue identified obesity as a major risk for cancer, and the food environment, especially high energy and low nutrient processed foods, as strong and prevalent risk factors for obesity. Further engagement highlighted challenges in the post-diagnostic setting, where similar opportunities to understand the complex interplay of nutrition, physical activity, and weight will inform better health outcomes.

Going forward, holistic research approaches, encompassing insights from multiple disciplines and perspectives, will catalyze progress urgently needed to prevent cancer and improve public health.

## Introduction

### Robert L. Strausberg (Fig. 1) and Fiona Reddington (Fig. 2)

Over the past half century, much progress has been made in improving treatments for patients with cancer. Yet, the mortality rate from cancer remains unacceptably high. Alleviating the mortality, morbidity, and impact on quality of life attributable to cancer is a global health priority of increasing relevance to countries across the economic spectrum.

**Fig. 1:**
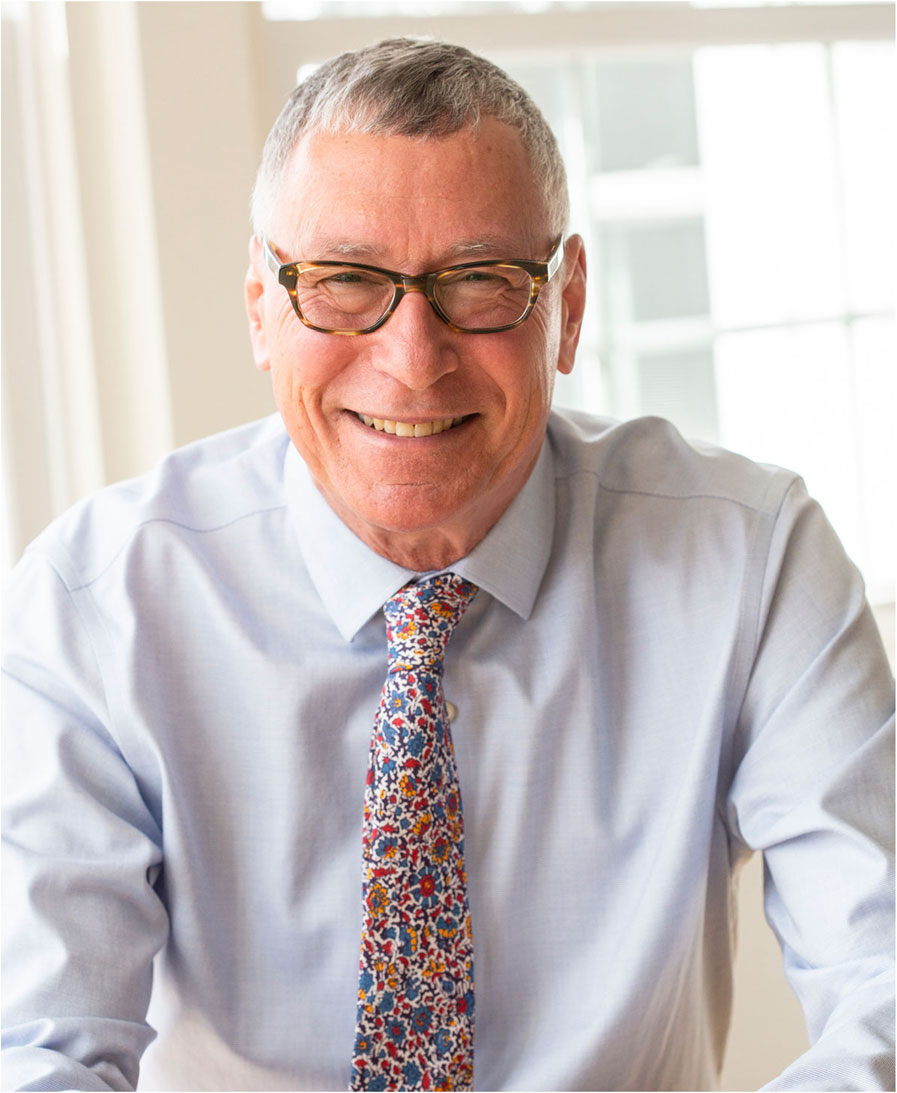
Robert Strausberg, PhD, is Deputy Scientific Director for the Ludwig Institute for Cancer Research. His scientific focus is on interdisciplinary approaches to improve the health of people worldwide through disease prevention and early intervention. Previously, he was Deputy Director of the J. Craig Venter Institute and served in leadership positions at the National Human Genome Research Institute, the National Cancer Institute, and the Institute for Genomic Research

**Fig. 2:**
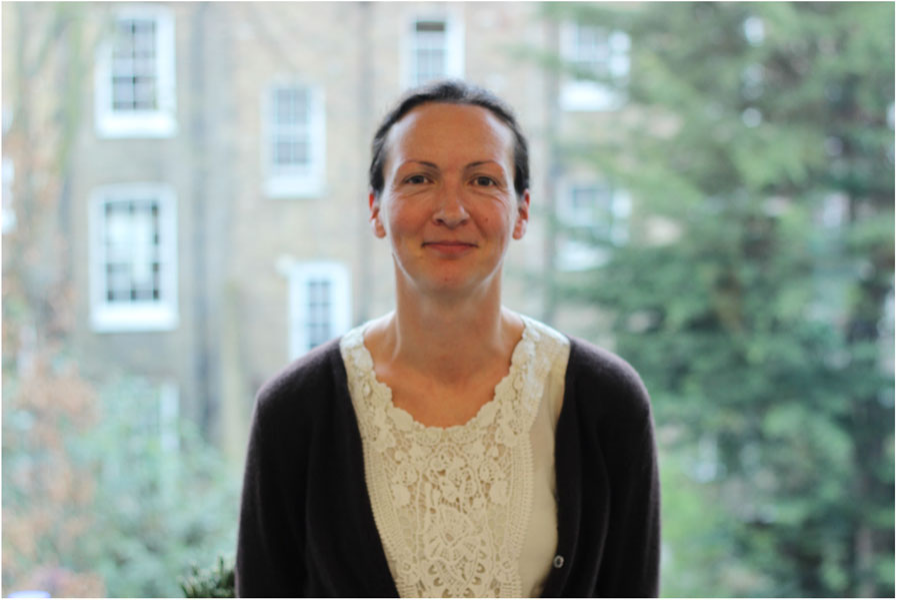
Fiona Reddington is Head of Population, Prevention and Behavioural Research Funding at Cancer Research UK and oversees the research portfolio in the areas of population research, prevention and early diagnosis. Following her BSc in Pharmacology and PhD in Neurophysiology, she went on to join the NHS as a project manager and subsequently moved into management roles at a national cancer network and the NCRI Informatics Initiative

Recent data suggest that approximately four in ten cancers are preventable through behavior changes alone [1, 2]. Public health measures have made progress in this area; these include prevention of lung cancer, which is largely addressed through smoking cessation, malignant melanoma, by limiting exposure to UV light, and cervical cancer by immunization against human papillomavirus. However, in the important area of diet, nutrition, and cancer prevention there is still much to learn about how to catalyze effective prevention efforts globally (see the EPIC study http://epic.iarc.fr/).

Having a shared goal of taking a radically new approach to cancer prevention, Ludwig Cancer Research and Cancer Research UK have sought to highlight the challenges and opportunities to enhance progress in this important area of public health research. A goal is to make this field a ‘go to’ area for the best and brightest scientists to perform research that will make for healthier people throughout the world.

In this spirit, Cancer Research UK and Ludwig Cancer Research convened an inaugural international Cancer Prevention and Nutrition Conference at the Francis Crick Institute in London on December 3–4, 2018. Discussions at the conference considered the field holistically, weaving together insights from multiple disciplines and perspectives. The sessions were organized into six distinct areas, which are important individually, but even more powerful when integrated. In this Forum article, the session chairs of the meeting have served as co-authors and prepared their sections in conjunction with the other speakers.

The meeting included much discussion about the challenges in defining effective prevention strategies based on nutritional and dietary change. Interdisciplinary research, incorporating disciplines such as business and marketing, political science, environmental sciences, geography, data and systems sciences, as well as simulation modeling, offers great promise.

Participants at the conference were encouraged to consider multiple risk factors within the context of cancer prevention. As the world continues to open – with improved mechanisms to share data, enhanced collaboration across continents, and cross-pollination increasing among traditional siloes – the links between nutrition and cancer prevention research are potentially more understandable and actionable. This provides an outstanding opportunity to invigorate conversations and stimulate the broadest spectrum of research talent to work in concert.

We hope that this Forum article will stimulate new approaches to cancer prevention research that are scientifically, methodologically, technologically, and internationally integrated and conducted with a clear sense of urgency to improve public health.

## Understanding dietary risks and cancer

### Walter C. Willett (Fig. 3) and Elio Riboli (Fig. 4)

#### Methodological issues

Interest in diet in the cause and prevention of human cancers was fueled in the 1970s and ‘80s by findings from ecological studies documenting large international differences in cancer rates that were correlated with per capita intakes of meat, fat, and other dietary factors [3]. In migrant studies, populations moving from low- to high-incidence regions developed cancer rates like those of long-term residents, indicating that these major differences were not due to population genetic factors. The effects of various dietary factors on tumorigenesis in animal models heightened attention to diet, but more detailed human studies were needed because the ecological correlations were potentially confounded by many nondietary variables. Since 1980, much attention has been devoted to methodological issues in the study of diet and human cancer, including study design, assessment of diet, and analysis of epidemiologic data on diet [4]. Most of the early studies of diet and cancer used case-control designs in which diet was assessed retrospectively after the diagnosis of cancer. Concerns about biases due to selection of participants and recall of diet have been proven to be justified because later results of prospective studies often did not support the earlier findings. Thus, prospective cohort studies, in which diet is assessed before the outcomes are known, have become the primary design.

**Fig. 3:**
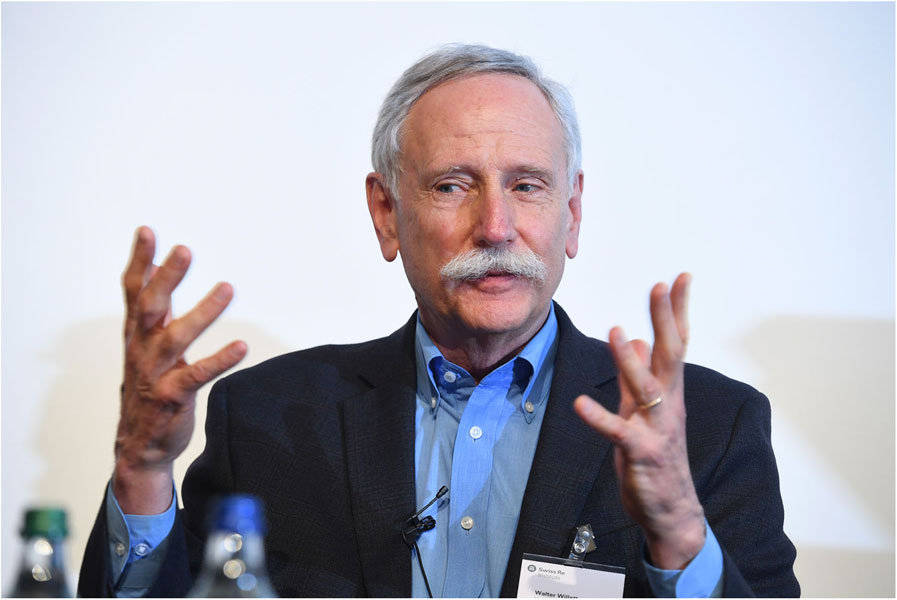
Walter Willett is Professor of Epidemiology and Nutrition at the Harvard T.H. Chan School of Public Health and Professor of Medicine at Harvard Medical School in Boston, Massachusetts. He served as Chair of the Department of Nutrition at Harvard T.H. Chan School of Public Health for 25 years. He has published over 2000 articles, primarily on lifestyle risk factors for heart disease and cancer, and has written the textbook *Nutritional Epidemiology*

**Fig. 4:**
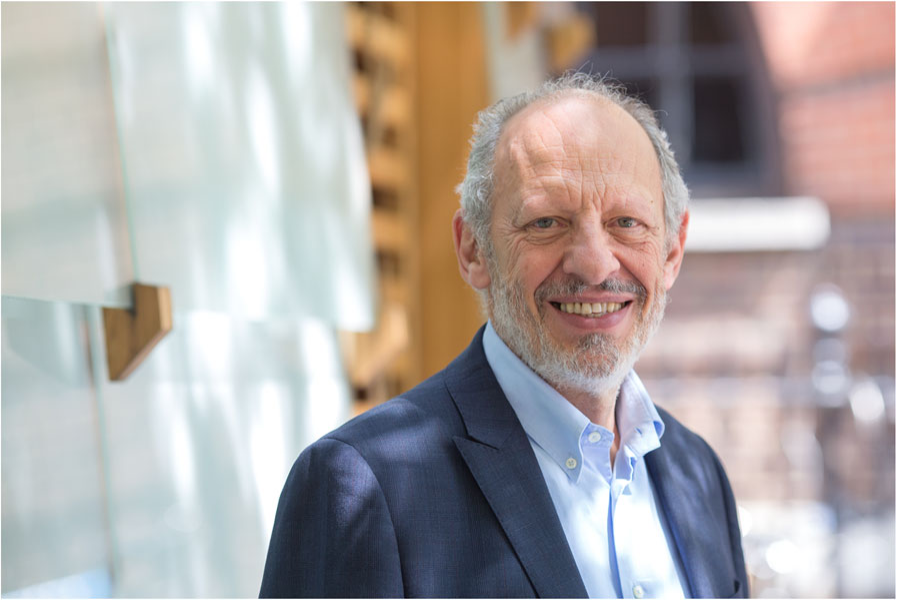
Elio Riboli holds an MD degree and an MPH from the University of Milan and a MSc in Epidemiology from Harvard University. In 1990, he initiated the European Prospective Investigation into Cancer and Nutrition, a large population-based cohort designed to investigate the role of diet, nutrition and metabolic factors in the etiology of cancer and other chronic diseases. In 2006, he moved to Imperial College, where he became the first Director of the Imperial School of Public Health (2008–2017) and continues his research in the field of nutritional epidemiology of cancer

Early analyses of day-to-day variation in dietary factors indicated that in order to measure the intake of an individual of most nutrients and foods, recall of consumption over a 24-h period would be required. [5]. Thus, various forms of food frequency questionnaires (FFQs) have become the most widely used method of dietary assessment in epidemiological studies. Whether FFQs have enough validity to detect important associations has been addressed in many studies using both biomarkers and detailed recording of diets as comparators [6]. These studies have documented adequate validity, particularly for energy-adjusted intakes, which are of primary interest because changes in nutrient and food intake must be made within narrowly constrained energy intakes. Validity is supported by robust and replicated findings for many dietary factors measured by FFQs as predictors of diabetes and cardiovascular disease. Biomarkers of diet can complement assessments of intake, but these also have limitations as they are not available since many aspects of diet are rarely measured repeatedly to capture long-term exposures and are usually influenced by multiple nondietary factors.

#### Overview of the role of diet in cancer causation

Beginning in the early 1980s, the possibility that diet could be a key link between what was generically called the ‘environment’ and cancer led to a new generation of very large cohorts of healthy volunteers who provided data on diet and other behavioral characteristics at baseline and were followed for cancer incidence and mortality. The World Cancer Research Fund started, in the mid-1990s, the first worldwide effort to systematically review and summarize the epidemiological evidence on diet and cancer, which materialized in the 1997 First World Cancer Research Fund Report, followed by subsequent reports in 2007 and 2018. Based on the aggregate of several thousand publications in more than 50 large population cohorts and several million study participants, diet during mid-life appears to have a rather weak association with subsequent cancer risk [7]. Even when there is substantial consistency between studies in different populations, as is the case for red meat and fiber-rich foods and colorectal cancer, the variation in risks between high and low consumption levels are relatively modest (e.g., risk ratio in the order of 1.2–1.5). However, the prospective nature of the cohorts and the baseline anthropometric measurements taken in many cohorts, as well as some assessment of physical activity, has provided convincing, and initially unexpected, evidence that overweight/obesity is associated with increased risk of many cancers while moderate or high levels of physical activity are associated with reduced risk. Therefore, from a simple view that particular foods consumed could be a major factor modulating cancer risk between individuals and across the world, two decades of research based on very large scale epidemiological cohort studies have led to the current understanding that it is in most instances the combination of the type of food, the level of regular physical activity, and the accumulation of body fat (or its distribution and/or the ratio between fat/lean tissue mass) that constituted the basic triad of important metabolic factors that influence cancer development.

The complexity of the metabolic factors modulated by diet/anthropometry and physical activity may be a contributing factor for the lack of support for several prominent food and cancer hypotheses in large prospective studies, rather than just inadequate dietary assessment. In addition, it could be related to the natural history of cancer, in which exposures may act early in life and decades before diagnosis. Until now, few studies have been able to examine these temporal relationships; filling these gaps should be a priority for future research. While awaiting the development of cohorts to address these gaps, much could be learned from the systematic analysis of unexamined data on diet and cancer that already exists; support for such collaborative efforts would be highly cost-effective.

## Interfacing laboratory, epidemiological, and other clinical studies – moving from links to cause

### Richard M. Martin (Fig. 5) and Edward L. Giovannucci (Fig. 6)

Large geographic and temporal variations in cancer incidence and survival point to important environmental determinants, and epidemiological and laboratory studies indicate a key role for dietary factors in cancer development and progression [8]. These considerations suggest substantial potential for the development of cancer prevention strategies. However, the continuing debate around dietary interventions and policies illustrates the challenges of generating robust causal evidence for action. The current nutrition and cancer evidence-base is largely observational and prone to confounding, reverse causality, and inaccurate exposure assessment, precluding confident causal inference. Further, the biological pathways underlying diet and cancer relationships are poorly understood, pre-clinical data only weakly reproducible [9] and, while randomized controlled trials are the gold standard, they are expensive, time consuming, often not feasible, and can only address a limited number of interventions in selected populations.

**Fig. 5:**
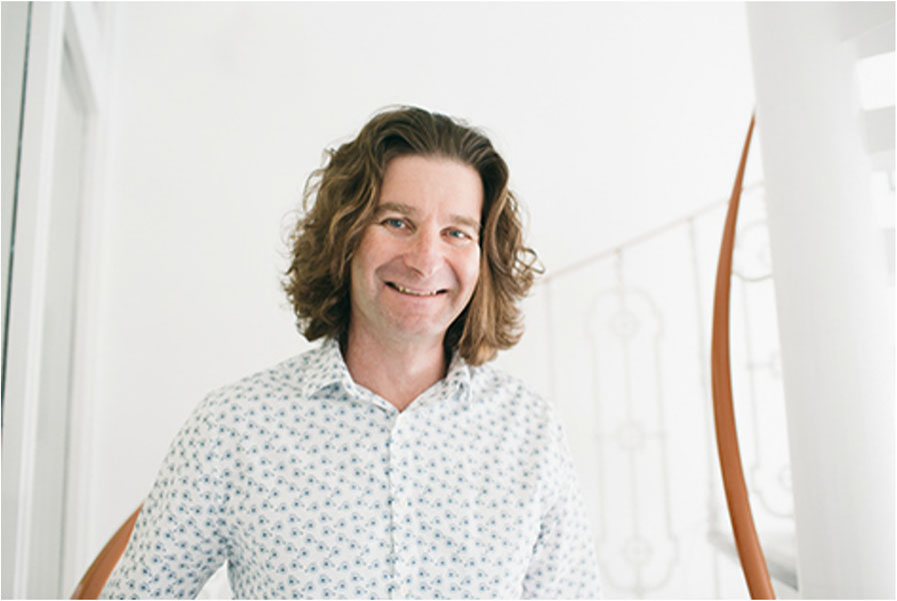
Richard Martin is a Professor of Clinical Epidemiology at the University of Bristol and Honorary Consultant in Public Health. His research interests include cancer epidemiology, the application of causal analysis methods to strengthen the evidence for developing primary and tertiary prevention interventions, and the identification of -omic biomarkers for secondary prevention

**Fig. 6:**
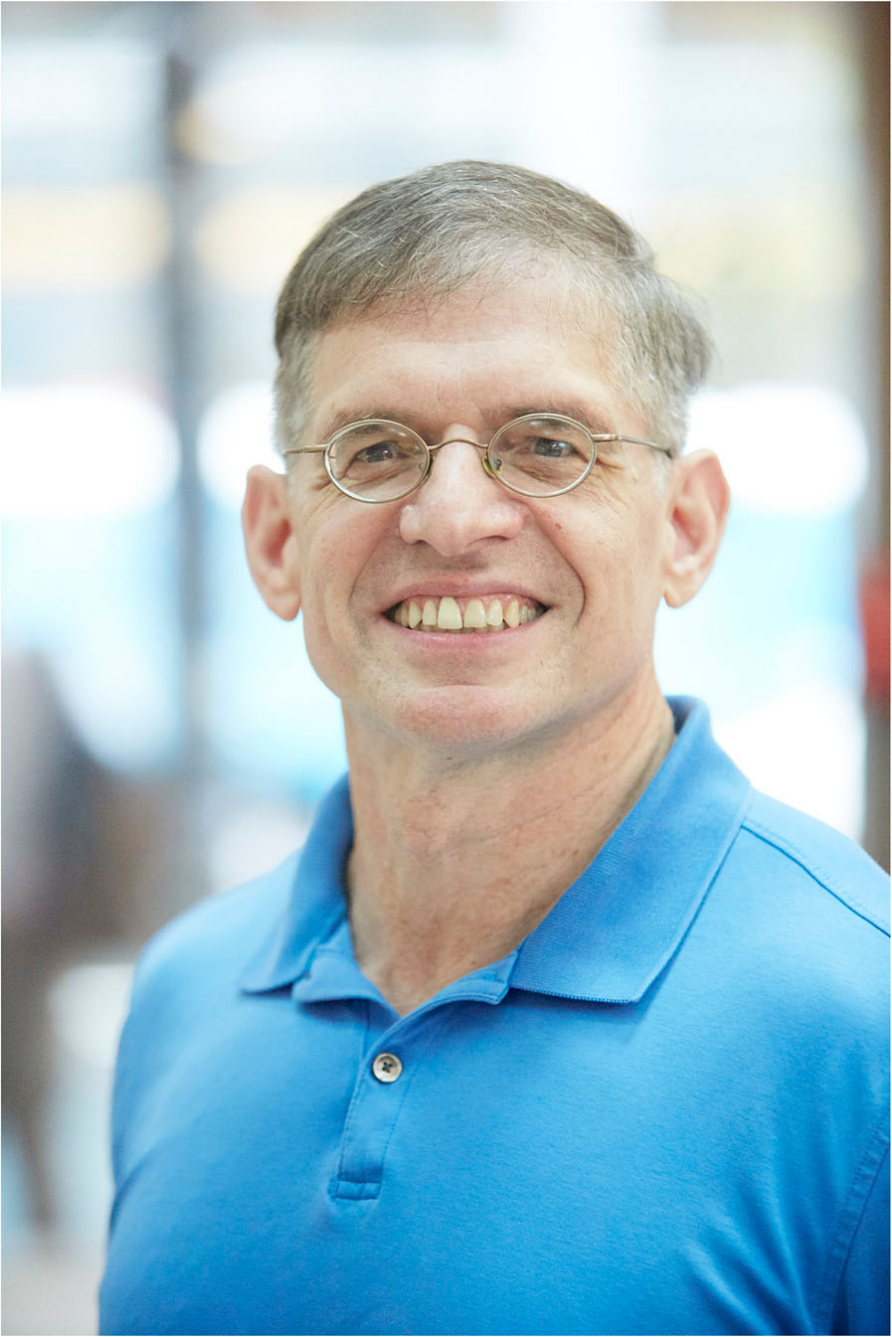
Edward Giovannucci graduated from Harvard University in 1980, received an MD from University of Pittsburgh in 1984, and then completed a doctoral degree in epidemiology from the Harvard T.H. Chan School of Public Health in 1992, where he is currently a Professor in the Departments of Nutrition and Epidemiology. His research focuses on how nutritional, environmental, and lifestyle factors relate to various cancers

#### How do we move from observational associations to causal links?

Recent advances in high-throughput assays to measure the genome, methylome [10–14], metabolome [15, 16], transcriptome [17], microbiome [18], and proteome [19], together with the creation of large research consortia and population biobanks, have vastly expanded the availability of high-dimensional molecular datasets from human samples, many of which are accessible through a range of bioinformatic platforms [20–22]. These studies have enriched our understanding of gene regulation and function [23] and provide an unparalleled resource for furthering our understanding of the causal underpinnings of cancer.

For example, with the surge in genome-wide association studies, Mendelian randomization has become firmly established as a major analytical tool in the causal understanding of cancer risk and mechanisms – robust germline genetic instruments from genome-wide association studies can now proxy thousands of cancer-related modifiable exposures and metabolic traits, and genome-wide data on tens- to hundreds-of-thousands of individual site-specific cancers are readily available [21, 22, 24]. In the field of epigenetics, we are now able to exploit recent advances in understanding the genetic architecture of DNA methylation variation across the human life course [14] to develop methods for investigating molecular mediation. DNA methylation-based biomarkers that reflect risk factors (rather than disease processes themselves) may provide a useful strategy to refine risk estimates and provide novel opportunities for risk-tailored screening and cancer prevention [25–28]. Indeed, the use of systemic molecular biomarkers, such as DNA methylation variation, may capture integrated biological effects of inter-related exposures (such as diet, physical activity and adiposity, and their downstream physiological consequences), and be more strongly associated with and predictive of cancer than focusing on single nutrient factors [29].

Promising advances in molecular epidemiological studies, the willingness of groups to collaborate to create large-scale consortia and the development of novel causal analytical methods, combined with our ability to readily integrate epigenomics, transcriptomics, metabolomics, and proteomics with data on hundreds of diseases, mean that we can readily map the influence of these molecular traits on complex human diseases, including cancer. In this way, we will more efficiently and robustly be able to rapidly prioritize potential interventions for independent replication or follow-up in experimental studies, based on robust causal analysis [30–32], deprioritize other targets, minimize deploying resources on non-causal targets [33, 34], predict unexpected effects (adverse and beneficial) of an intervention [35], validate exploratory analyses from clinical trials [36], and identify potential biological mechanisms underpinning exposure-cancer associations [30–32].

Evidence will continue to evolve with the complexity of changing exposures and population characteristics. Thus, a dynamic approach to measuring the entirety of the evidence from different sources, assessing outcomes of policies or natural experiments, and continuing surveillance are still needed. Nevertheless, the improved characterization of exposures, mechanisms, and biological understanding at the population level can contribute to strengthening confidence in preventive interventions.

## Metabolic health – dietary, genetic, and epigenetic factors

### Marc J. Gunter (Fig. 7) and Hal Drakesmith (Fig. 8)

The reprogramming of energy metabolism, one of the hallmarks of cancer and metabolic transformation, is a key event in tumorigenesis. The importance of metabolic dysfunction in driving cancer is supported by both observational and experimental evidence. For example, epidemiological studies have shown that obesity increases the risk of developing at least 12 different types of cancer [37], while data from both human studies and animal models have demonstrated that mutations in cellular pathways linking nutritional status and growth are frequently mutated in cancer (e.g., mTOR, PI3K). Currently, we do not fully understand the biology that connects metabolic dysfunction with cancer development at either a cellular or systemic level. However, accumulating evidence from multiple arenas of research are beginning to suggest some key links for how the overall metabolic state of the individual may drive molecular mechanistic alterations that underlie or accelerate carcinogenesis. Established mechanisms include obesity-associated alterations in systemic hormones and growth factors (particularly insulin and insulin-like growth factor-1, leptin, and sex steroid hormones), which induce pro-cancer signals in preneoplastic or neoplastic cells through their respective receptors [38–40]; cytokines and other inflammatory molecules are also altered and signal through their receptors and downstream signaling pathways.

**Fig. 7:**
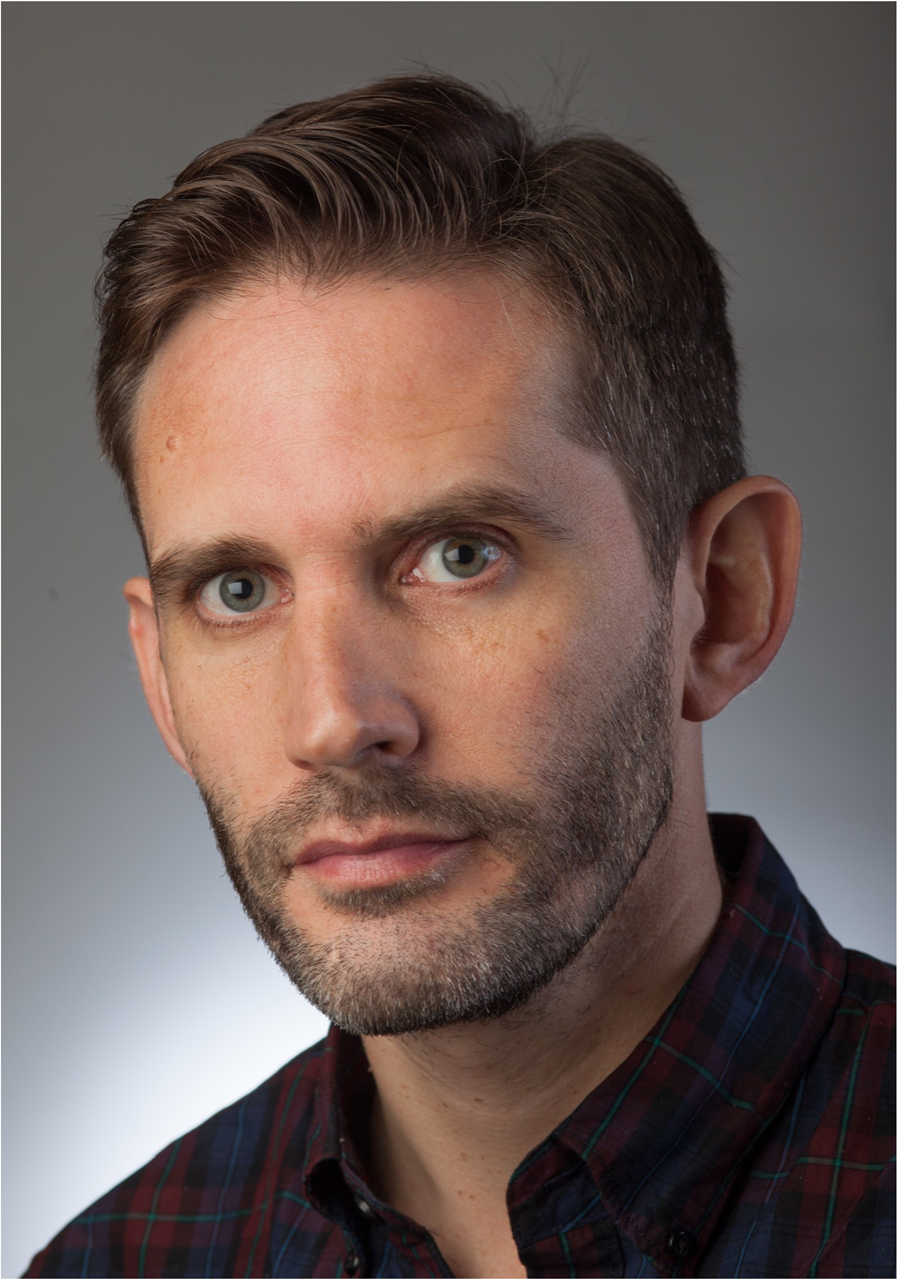
Marc Gunter is Head of the Section of Nutrition and Metabolism at the International Agency for Research on Cancer, the specialized cancer research agency of the World Health Organization. Dr. Gunter’s research focuses on the role of nutrition, diabetes, and obesity in the natural history of cancer, with an emphasis on metabolic dysfunction and in particular the insulin/IGF/mTOR pathway. He is principal investigator of a number of studies applying high dimensional metabolic profiling within the framework of large prospective and clinical cohorts, as well as intervention studies to identify novel biochemical pathways involved in cancer development and prognosis

**Fig. 8:**
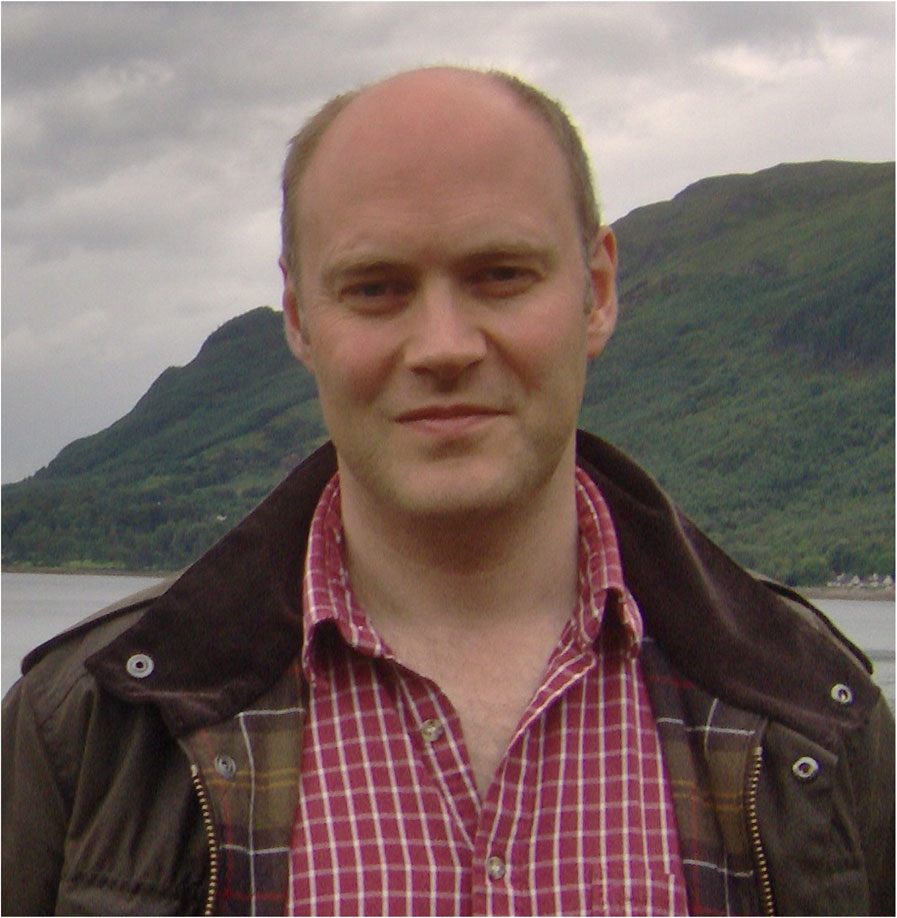
Hal Drakesmith was trained at the University of Cambridge, University of Kyoto, and University College London before moving to the University of Oxford. His laboratory in the MRC Human Immunology Unit at the Weatherall Institute of Molecular Medicine works on the interaction of iron homeostasis with immunity, metabolism, anemia, and inflammation

Mitochondria govern cellular energy metabolism, and analyses of gene expression profiles of cancer patients from The Cancer Genome Atlas revealed that mitochondrial genes are suppressed in cancers with poor clinical outcomes [41]. Further, mutations in key metabolic enzymes in the mitochondria, such as fumarate hydratase, succinate dehydrogenase, and isocitrate dehydrogenase, can cause cancer. For example, cells lacking functional fumarate hydratase accumulate fumarate, which in turn activates a plethora of biological processes linked with tumorigenesis [42].

Some cancer cells have increased uptake of certain nonessential amino acids, such as serine and glycine, which support maximal cancer cell proliferation [43]. Serine and glycine contribute to the synthesis of nucleotides (both DNA and RNA), proteins, and the antioxidant glutathione [44]. While nonessential amino acids can be synthesized de novo, diet is also an important source of these amino acids.

Together, these findings suggest that dysregulated metabolism can work in parallel to canonical oncogenic cascades to drive cancer, and that dietary input is a contributing factor to supplying the needs of altered cancer cell metabolism. In several models for cancer, dietary restriction of serine and glycine can slow tumor growth, increase survival, and potentiate the activity of anticancer drugs [45]. Therefore, targeted dietary interventions may be a viable way to enhance cancer therapy and improve survival in some cancer patients.

An interesting link for how diet might influence cancer development is through the interface of gut microbes, the metabolites they produce, and the immune system. Within the gut, the microbiota and the immune system regulate intestinal function and interact with dietary components to maintain tissue function. In particular, a population of immune cells called regulatory T cells, arises in the intestine and their differentiation is driven in part by specific members of the commensal microbial community and their metabolic products [46]. These cells in turn regulate the composition and metabolic status of commensal microbiota and maintain a fine balance between immunity and tolerance in the gut, whilst also restraining precancerous lesions in the gastrointestinal tract.

Overall, these studies demonstrate new links between altered cellular metabolism, often driven by mitochondrial dysfunction, interacting with specific dietary components to produce an imbalance of metabolites within a cell that can promote tumor growth. Systemic adiposity contributes to the development of some cancers, potentially through gut-oriented processes that affect intestinal microbiota and immune activity, which in turn influence inflammation and energy balance. In terms of preventative measures, a low body fat state is protective against certain cancers. However, understanding how particular dietary factors, biochemical processes, and metabolites interact with microbial, immune, and other environmental factors to generate cancer risk remains a considerable challenge. Nevertheless, the rationale that dietary and metabolic interventions have a role to play in preventing cancer is increasingly supported from a firm mechanistic basis.

## Developmental origins of cancer

### Karin B. Michels (Fig. 9) and Robert A. Waterland (Fig. 10)

Environmental influences during critical developmental periods can have a long-lasting impact on the risk of many diseases, including cancer. The study of such long-term effects of early exposures is broadly referred to as the developmental origins of health and disease (DOHaD). Animal and human population-based studies indicate that, during prenatal and early postnatal life, exposures such as through nutrition, radiation, infection, pharmacologic exposures, and environmental contaminants can set the stage for increased cancer susceptibility later in life.

**Fig. 9:**
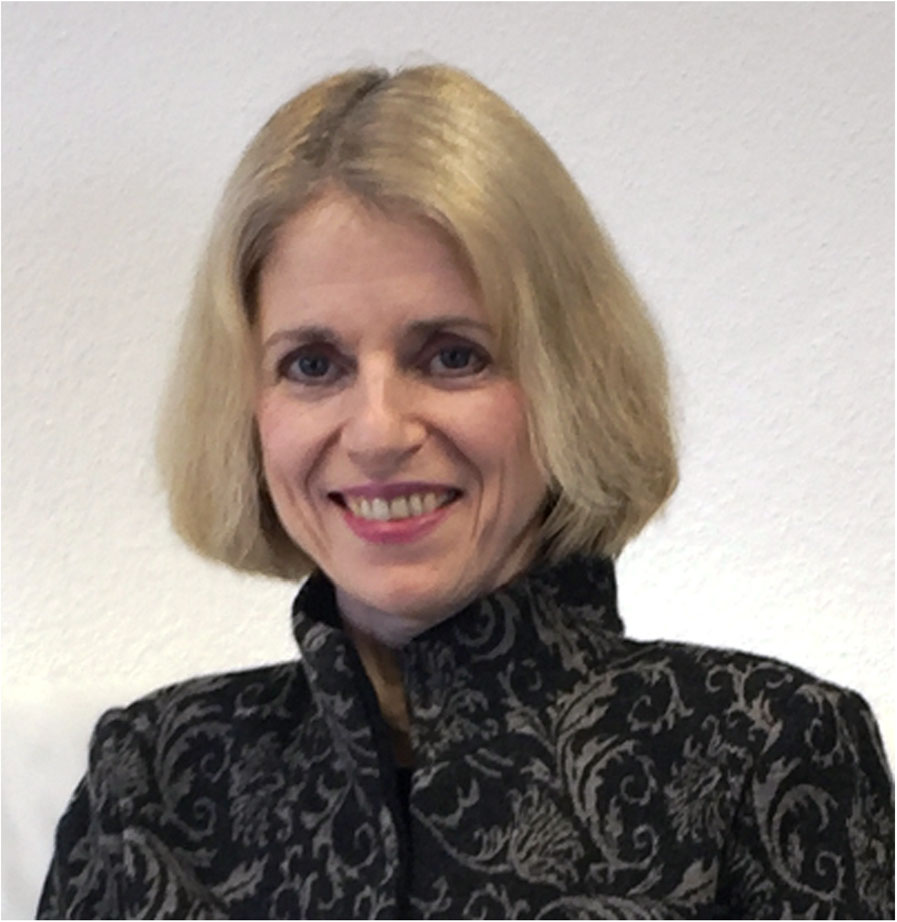
Karin B. Michels, ScD, PhD, is Professor and Chair of the Department of Epidemiology at the UCLA School of Public Health in Los Angeles, California. Her research interests include cancer, nutritional, and epigenetic epidemiology. She has made seminal contributions to elucidating the early life origins of cancer, specifically breast cancer

**Fig. 10:**
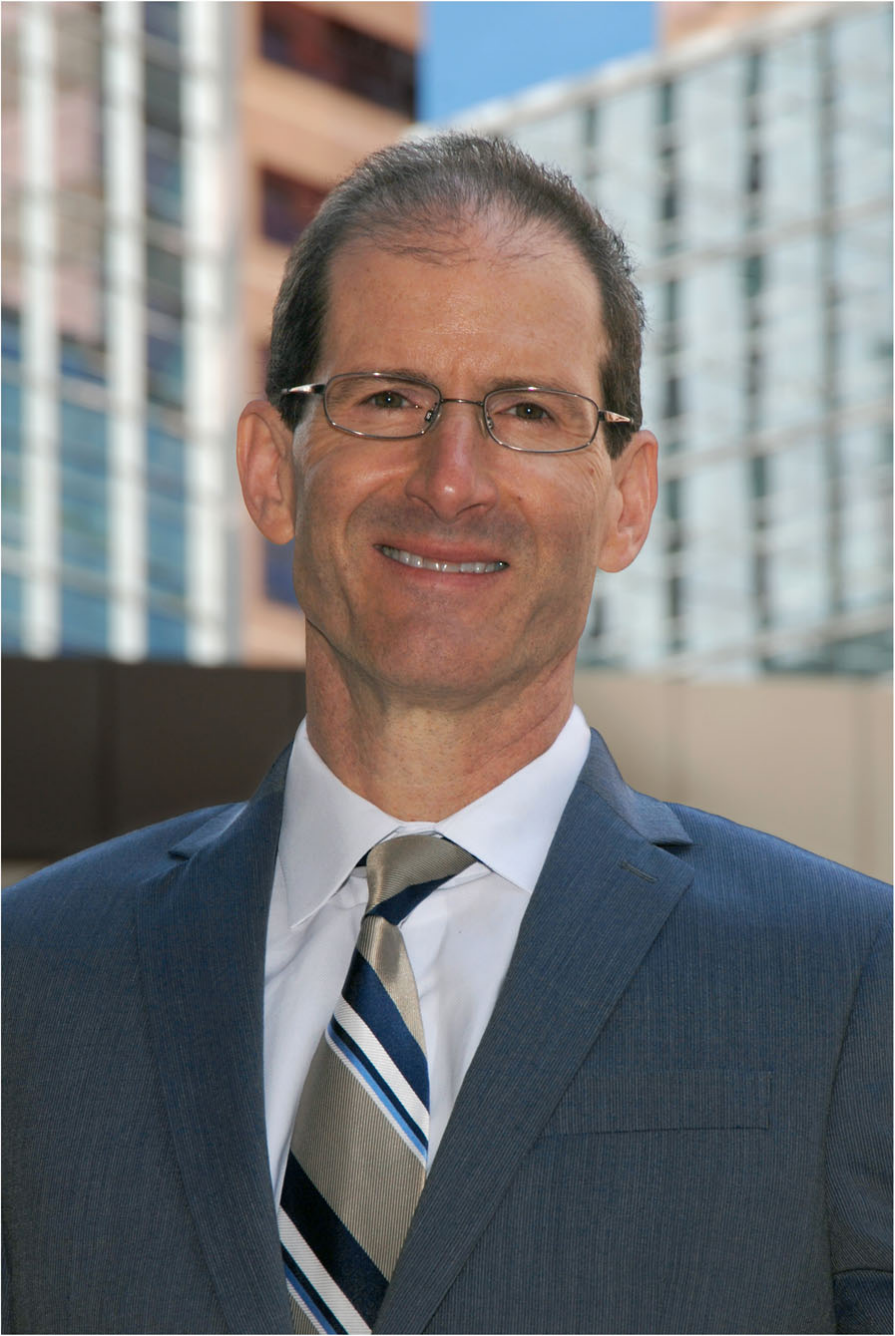
Robert A. Waterland, PhD, is a Professor of Pediatrics and Molecular and Human Genetics at Baylor College of Medicine in Houston, Texas. His research focuses on understanding epigenetic mechanisms in the developmental origins of health and disease

Available evidence for the developmental origins of cancer is strongest for some types of cancer. The best studied cancers with respect to early life influences are breast cancer, leukemia, and uterine cancer. For breast cancer, high birthweight, high birth length [47, 48], early age at peak growth [49] and early menarche [50] (all potential indicators of early overnutrition and exposure to growth factors), as well as early exposure to radiation [51] and low body fatness in childhood [52] are all associated with an increased risk of later breast cancer. Maternal pre-eclampsia [48] and caloric restriction during adolescence and early adulthood [53] are associated with reduced risk of later breast cancer. High birthweight has also been associated with elevated risk of several childhood and other adult cancers [54–56]. Other types of cancer have been linked to more specific early exposures, such as paternal pre-conceptional smoking to childhood leukemia [57], childhood infections to Hodgkin’s and non-Hodgkin’s disease [58], infections during pregnancy to leukemia [59], and factors affecting immune development at birth to pediatric leukemias [60]. Intrauterine exposure to diethylstilbestrol has been linked to increased risk of developing adenocarcinoma of the vagina [61].

Clearly, if a considerable proportion of lifetime cancer risk is determined by environmental factors during early life, understanding cancer from a DOHaD perspective may provide new opportunities for cancer prevention. Identifying the windows of susceptibility during early life, narrowing down the most important modifiable risk factors that shape cancer predisposition, unravelling relevant mechanisms, and understanding why some cancers are particularly sensitive to early influences are essential steps to move this agenda forward. Parsing the attributable fraction of cancer type-specific risk factors, including diet, physical activity, obesity, infection, radiation, and environmental chemicals, at various points across the life course will enhance our understanding of cancer etiology and inform prevention strategies. Optimizing life choices, such as diet, may need to take into consideration balancing the risk of other conditions such as cardiovascular disease or diabetes.

Two major barriers to progress are (1) the long period of time between the early exposure and the resulting cancer – up to several decades – and (2) our limited understanding of the fundamental biological mechanisms mediating these long-lasting effects [62]. Regarding the first, in our view, initial efforts focused on understanding the developmental origins of childhood cancer should be a top priority; this would allow the design of studies spanning several years rather than decades [63]. Further, the pooling of existing cohort data with relevant exposure data and available follow-up data for cancer should be a key effort. To the extent that the developmental origins of childhood and adult cancer share mechanistic features, knowledge gained from studies of childhood cancer will help refine DOHaD studies of adult cancers.

Regarding the second barrier, there are emerging opportunities to advance our understanding of the role of epigenetic and other mechanisms in the developmental origins of cancer. The diseases most commonly studied with respect to DOHaD are hypertension, cardiovascular disease, obesity, and type 2 diabetes; relatively few studies have focused on DOHaD and cancer. This is surprising, given that epigenetic mechanisms are currently the most studied mechanisms postulated to explain the long-term persistence of developmental programming [64] and cancer is, by far, the most studied (and best understood) chronic disease with respect to epigenetic etiology. The stage is set for rapid advances in epigenetic mechanisms mediating the developmental origins of cancer if more research can be stimulated in this area. A major hurdle facing such studies is the cell type-specificity of epigenetic markers [64]; yet, recent studies have made progress identifying human metastable epialleles, which exhibit systemic interindividual variation in DNA methylation [65], and initial studies are showing promise using these as markers of cancer risk [66].

Longitudinal data collection with real-time exposure assessment, biospecimen collection, and endpoint ascertainment remain challenging but are urgently needed. Alternatively, retrospective studies exploiting documented or manifested exposures such as clinical records and/or previously collected specimens (e.g., newborn blood spots or Guthrie cards) can be useful. A novel recent investigation using Guthrie cards suggested that pregnancy levels of androgens were associated with testicular tumors that developed in adolescents but not among infants [67]. Guthrie cards are additionally useful for measuring immune development [68], nutrition [69], and DNA methylation [70]. Rather than waiting for cancer to manifest, it may be feasible to identify intermediate endpoints that can be utilized as markers of future cancer risk. Identification of such intermediate markers will benefit from consideration of molecular and cellular aspects of carcinogenesis. There are many opportunities to apply mechanistic insights from animal model studies to refine population-based analyses.

Prevention efforts may start prior to conception by targeting gametes and preventing epigenetic errors, exploring what represents an optimal pregnancy diet, and evaluating neonatal screening for acquired genetic mutations and congenital cytomegalovirus infection. Whether vaginal birthing may affect cancer susceptibility via modulation of the microbiome and the innate immune function remains to be explored in both animal and population-based studies. It remains unresolved whether cancer screening and active prevention is possible for cancers like leukemia. Risk stratification based on genetic predisposition represents another sensible yet underexplored avenue for early intervention to reduce future cancer risk.

Stored biological samples and existing clinical data suggest outstanding opportunities for multidisciplinary teams to test associations, understand underlying mechanisms, and design interventions. However, funding opportunities are sparse, and resources are reluctantly applied to prevention, let alone early life factors that are decades removed from cancer occurrence. We hope our pressing call to action will be heard.

## Diet after a diagnosis of cancer

### Rebecca J. Beeken (Fig. 11) and Edward L. Giovannucci (Fig. 6)

There is increasing evidence that, in addition to playing a role in cancer development, diet may influence outcomes after cancer diagnosis [71]. However, the current recommendations for survivors are based largely on extrapolation from cancer prevention recommendations. Dietary survivorship studies encompass a variety of settings, including general nutritional support for advanced stage cancer patients, specific dietary factors or nutrients that may improve treatment responses and survival for specific cancers, and the influence of nutrition for overall mortality with cancers that have a good prognosis for long-term survival and for which the main causes of death will not be related to the cancer itself; the issues for each of these vary. There is an urgent need for carefully designed and adequately powered cohort studies and randomized interventions with appropriate outcomes for each phase of survival across cancer types. While large scale interventions may ultimately provide the strongest evidence, rigorous assessment of observational data is essential to justify them.

**Fig. 11:**
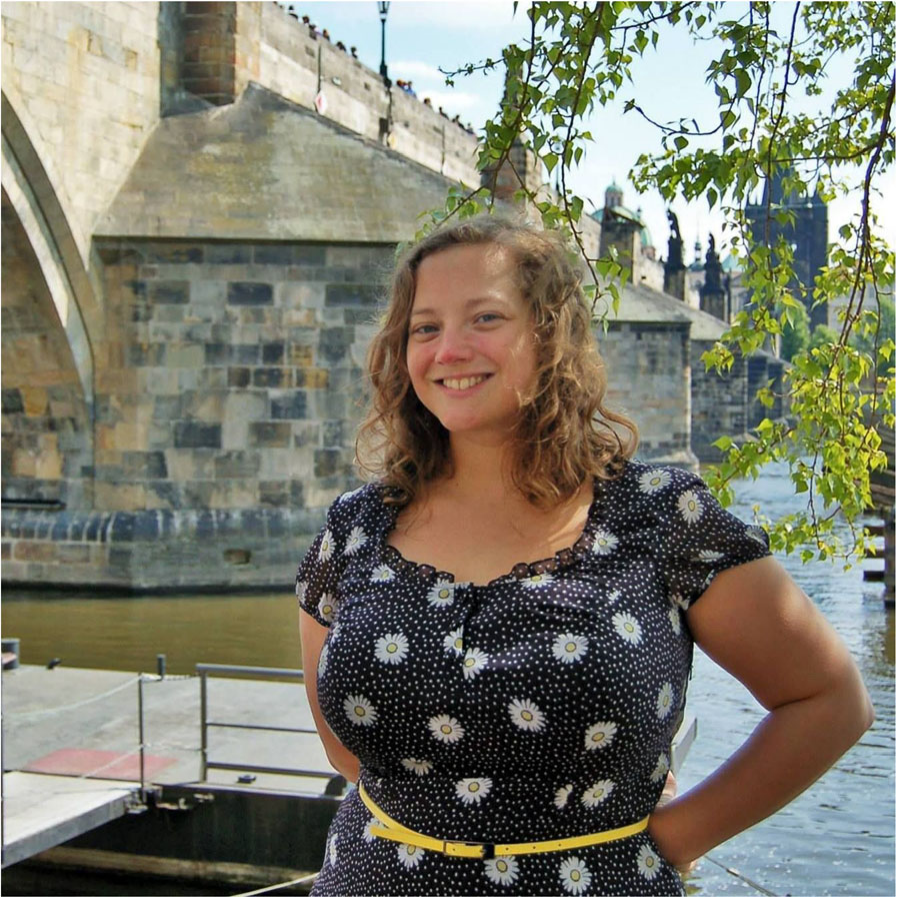
Rebecca J Beeken, PhD, is a tenure-track Yorkshire Cancer Research University Academic Fellow at the University of Leeds. Her academic background is in behavioral science and health psychology, and her primary research interest is in behavior change for cancer prevention and control. Rebecca currently co-leads a Cancer Research UK-funded program of work exploring the efficacy of a brief, habit-based intervention for improving dietary and physical activity behaviors in people living with a diagnosis of cancer

Beyond the typical challenges of observational studies of diet and nutrition and cancer incidence, survivorship studies have additional challenges. First, any effects could differ by cancer stage, cancer subtypes, and by treatment, which could all influence survival. Thus, the recommendations for survivorship may need to be more nuanced than those for primary prevention. Careful consideration needs to be given to how best to communicate these complex, and potentially changing, messages to healthcare professionals, patients, and the public. Secondly, because the study population already has cancer, the potential for reverse causation for observed associations is substantial. For example, higher levels of physical activity post-diagnosis appear to be associated with better survival for some cancers, but a possibility may be that patients who become progressively sicker, and hence have a poorer prognosis, may be less able to exercise. Finally, behaviors in the post-diagnostic period may correlate with those in the pre-diagnostic period, so if an association is observed in the post-diagnostic period, the causal effect could be occurring in the pre-diagnostic period. Thus, an intervention based on such observations, if begun in the post-diagnostic phase, may not confer the benefit suggested in the observational study. Studies combining primary prevention with post-diagnostic work may offer unique opportunities to explore the impact of interventions across the cancer continuum.

There are also practical challenges for studies that are specific to the post-diagnosis setting. In particular, the support of healthcare professionals is crucial for recruitment of patients and implementation of any successful intervention. Healthcare professionals can act as gatekeepers, limiting patient access to interventions and biasing recruited samples. The challenge of engaging clinicians who face a number of competing time demands and have concerns around the effectiveness of an intervention, as well as patient blaming, their perceived lack of interest, and them being too ill to participate, needs to be addressed [72, 73]. Strategies are needed that address the range of barriers to participation in, and adherence to, interventions among people with a diagnosis of cancer, especially hard-to-reach patients [74–76], to ensure existing inequalities in outcomes are not increased.

Additionally, there is a need to better understand mechanisms of action of interventions and to untangle the complex interplay of nutrition, physical activity, and weight on any observed effects; it is inadequate to study nutritional factors in isolation. Furthermore, developing interventions that not only change behavior but also support individuals to maintain those changes over the longer term represents a significant challenge [77]. Theories that specifically address maintenance, such as habit-formation theory [78], may offer useful starting points, but have not been widely tested [79]. Research to identify the optimal triangulation of dose, modality and timing of interventions, and the extent to which interventions can and should be personalized is also needed. Digital technologies and the use of artificial intelligence may offer new, more efficient means of tailoring interventions.

To date, dietary intervention studies have predominantly focused on survivors of common cancers (breast, colorectal, and prostate) who have completed treatment. However, there is increasing interest in developing interventions for delivery during the post-diagnosis but pre-treatment stage (i.e., prehab) [80], and for patients facing recurrence or with incurable disease [81]. The feasibility of integrating such interventions into standard care needs to be examined and identifying appropriate outcome measures is crucial. Studies assessing the effect of interventions on patient-reported outcomes, quality of life, and symptom management, as pre-planned, primary endpoints are lacking [82]. Recent studies have begun to explore potential interactions between nutrition and treatment [45] and the role of the microbiome and immune system via biomarker trials [83, 84]. Results from ongoing trials exploring the impact of behavioral interventions on cancer-relevant outcomes are also eagerly awaited, but these require substantial efforts. There is also a call to make better use of existing data and to promote the collection of nutrition data from patients as part of standard care to support this endeavor going forward.

## The food environment and cancer prevention

### Linda Bauld (Fig. 12) and Hilary J. Powers (Fig. 13)

Considerable progress has been made in understanding the causal links between diet, nutrition, physical activity, weight, and cancer incidence. Research findings have informed guidance on cancer prevention from national and international organizations [7, 85]. These guidelines commonly provide information on healthier diets, maintaining a healthy weight, avoiding sedentary behavior, engaging in physical activity, and avoiding alcohol. There is good evidence that adhering to cancer prevention recommendations is associated with lower risk of some cancers [86, 87]. However, there are well-acknowledged obstacles that individuals and communities face in adhering to cancer prevention recommendations and attention needs to be focused on addressing the wider social and commercial determinants that affect individual capability, opportunity, and motivation to engage in healthy behaviors [88, 89]. In particular, inadequate attention has been paid to food environments, notably the pricing, promotion, and availability of different types of food and the content, formulation, packaging, and labelling of products [90]. Also described as the ‘foodscape’ [91], food environments include the physical environment, which is particularly relevant when considering overweight and obesity as a risk factor for cancer.

**Fig. 12:**
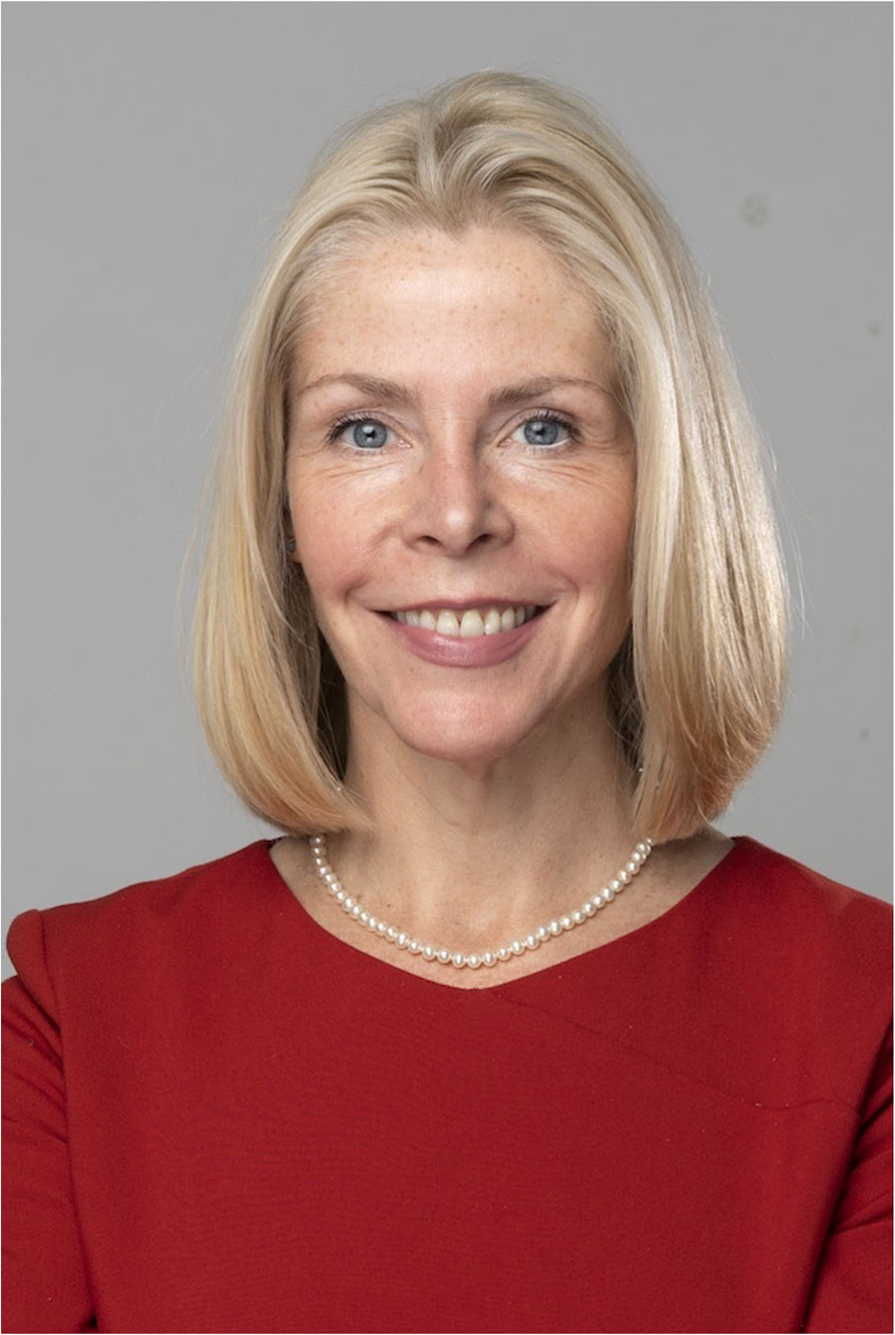
Linda Bauld is the Bruce and John Usher Professor of Public Health at the University of Edinburgh, Deputy Director of the UK Centre for Tobacco and Alcohol Studies, and holds the CRUK/BUPA Chair in Cancer Prevention at Cancer Research UK. She is a behavioral scientist with a particular interest in the primary prevention of cancer and has conducted a range of studies to inform or evaluate policies and programs to address tobacco, alcohol, and overweight and obesity. She is a former scientific adviser on tobacco control to the UK government and is a member of a number of policy and research funding committees in the UK, Canada, and Europe

**Fig. 13:**
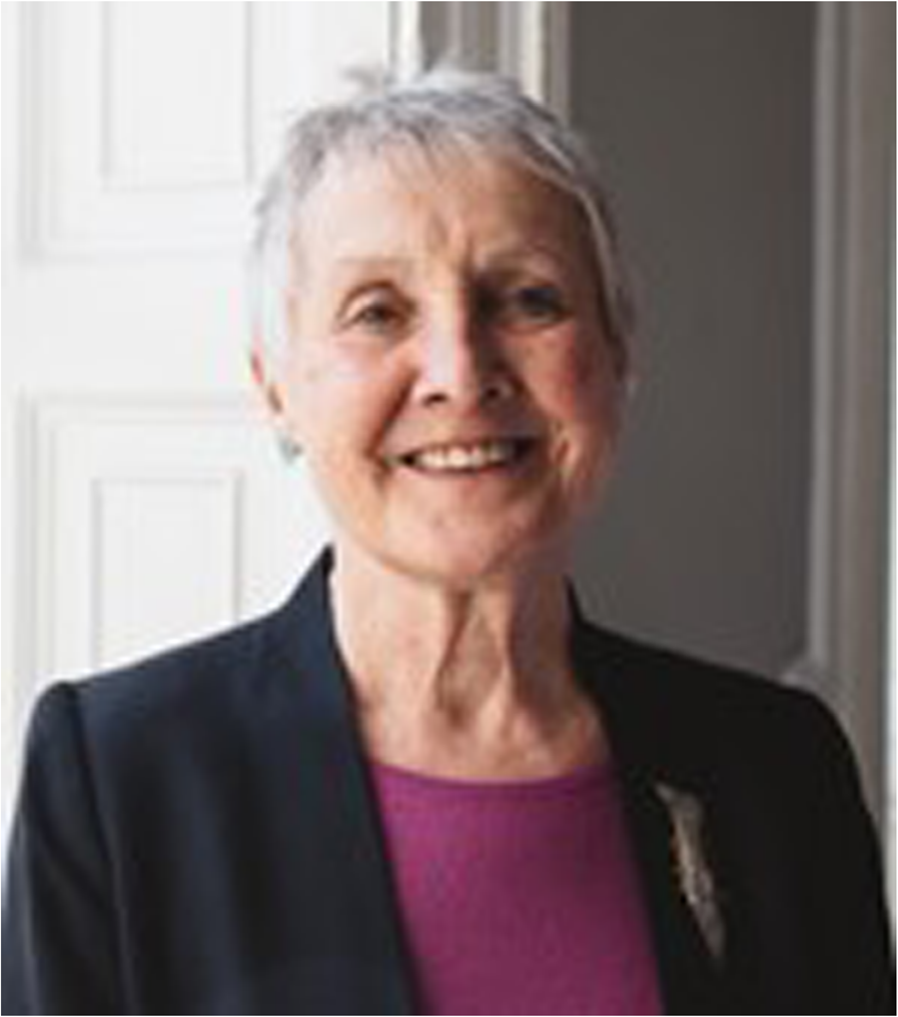
Hilary Powers is Professor Emerita at the University of Sheffield, UK. Her research has focused on micronutrients and cancer risk, at the epidemiology and molecular level. She has worked extensively with World Cancer Research Fund International on their reports into food, nutrition, physical activity, and cancer prevention

The consumption of high energy and low nutrient processed foods is a strong and prevalent risk factor for overweight and obesity and reducing the intake of these products is relevant for cancer prevention [92]. There is growing evidence that marketing and promotion affects dietary choices, including among children; exposure to high energy and low nutrient processed food advertisements on television and increasingly online has been shown to increase energy intake [93, 94]. The availability of these types of foods also has a role to play. For example, studies have identified a positive association between the density of unhealthy food outlets in a neighborhood and the prevalence of overweight and obesity in children [95, 96]. Location of high energy food items by tills in retail outlets can also influence choice [97, 98]. The relationship between deprivation and poor diet also calls for a greater focus on the food environment. Studies in a number of high-income countries, including the UK, have found that there is a clustering of fast food outlets in areas of deprivation along with more limited access to retail outlets that provide a wider range of healthier food options [99, 100].

Changing the food environment is possible and doing so could make an important contribution to the prevention of cancers linked to dietary risk factors and overweight and obesity. Action by retailers and manufacturers can contribute, as recently demonstrated by some success in voluntary reformulation to reduce salt in a range of products, and in the removal of confectionary from supermarket checkout aisles [98]. Arguably, however, population level policies introduced by governments have the greatest role to play. There is promising evidence that fiscal policies, including taxes on sugar-sweetened beverages, can reduce consumption of these products [101, 102]. Restrictions on marketing, including the timing, content, and extent of advertising, could affect consumption, particularly by children, and help shift social norms away from prevalent dietary choices that favor high energy and low nutrient processed foods. Greater attention should also be given to the local policy context and the importance of spatial planning for health. Local planning tools should be used to limit the growth and proliferation of unhealthy food outlets [103]. Planning decisions should also give greater consideration to the provision of opportunities for increased physical activity. There is evidence from different countries that planning decisions that provide useable green spaces in urban settings, a better cycling and walking infrastructure, and accessible public transport options can be effective in increasing physical activity [104].

Research to inform the development of policies and interventions to improve the food environment and prioritize cancer and other noncommunicable disease prevention efforts requires interdisciplinary collaborations between researchers with skills in nutrition, behavioral science, economics, geography, epidemiology, and other disciplines. Researchers need skills, not only in identifying appropriate context-specific methods and analysis, but also in knowledge translation and in engaging policymakers and the public. Support to identify appropriate frameworks and circumstances for collaboration with industry are also required, along with skills to understand and address conflicts of interest, both in the conduct of research and the availability and interpretation of often conflicting sources of evidence [105, 106]. Food systems and the food environment are complex and adaptive and research to better understand and improve them should to be similarly responsive [107].

## Conclusions

### Jessica Brand (Fig. 14) and Rachel A. Reinhardt (Fig. 15)

Ludwig Cancer Research and Cancer Research UK are privileged to have brought together world leaders in the field for a holistic discussion of the current challenges and opportunities in nutrition and cancer prevention. As discussed in this Forum article, strong themes emerged from the conference, consistently highlighting that obesity prevention, nutrition, and physical activity play a major role in worldwide cancer prevention. Many factors impact on progress with respect to these themes, which will be best served through integrated and systematic research. These include, for example, different experiences in childhood and the physical, social, economic, and digital environments that we inhabit.

**Fig. 14:**
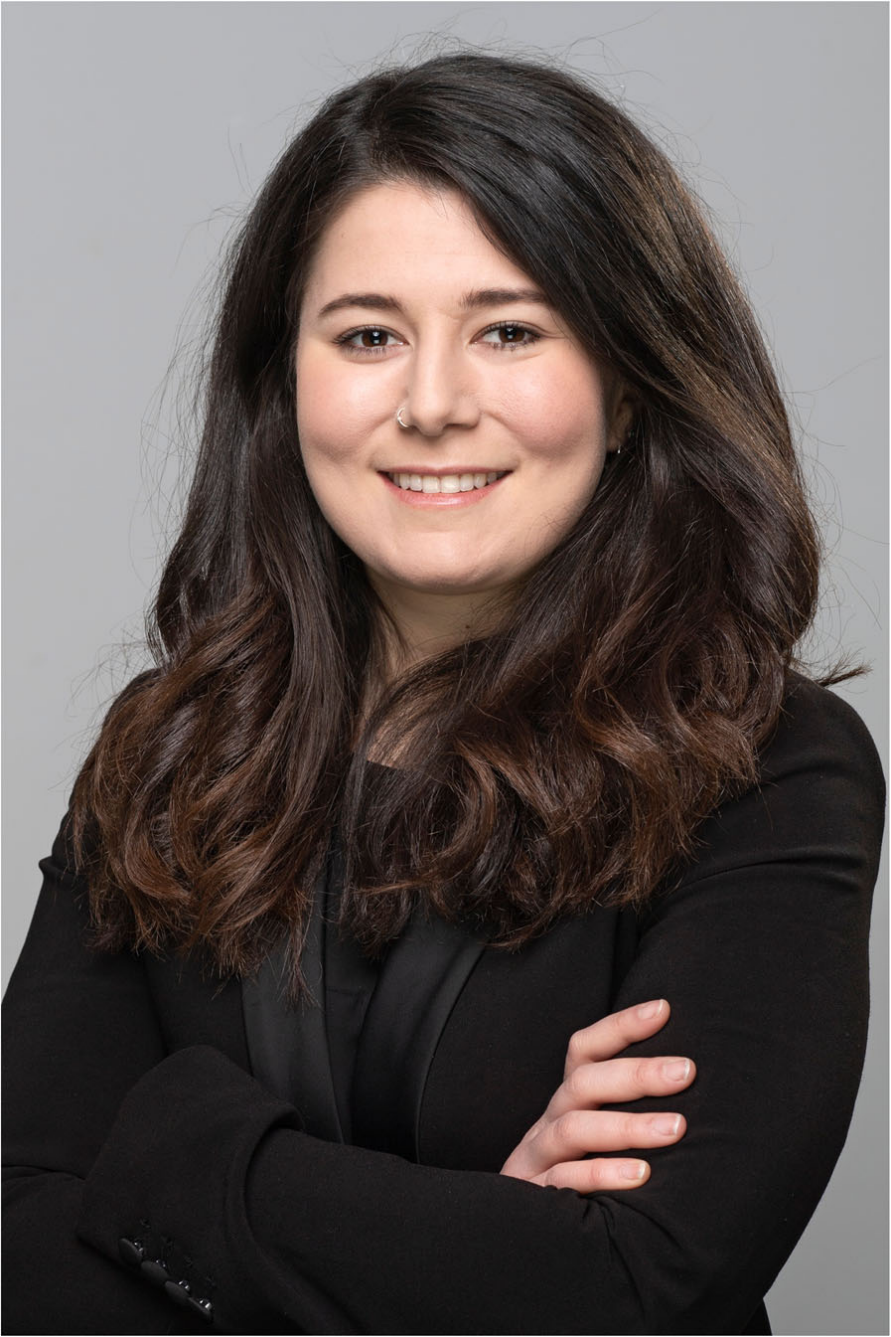
Jessica Brand is a Research Funding Manager in the Population, Prevention and Behavioural Research team at Cancer Research UK (CRUK) with a specific focus on the prevention elements of the portfolio. Having completed a BSc in Chemistry, she worked in academic journal publishing at the Royal Society of Chemistry, before moving onto project managing small researcher support grant schemes. Other roles in grant management followed, and she has been with CRUK since August 2017

**Fig. 15:**
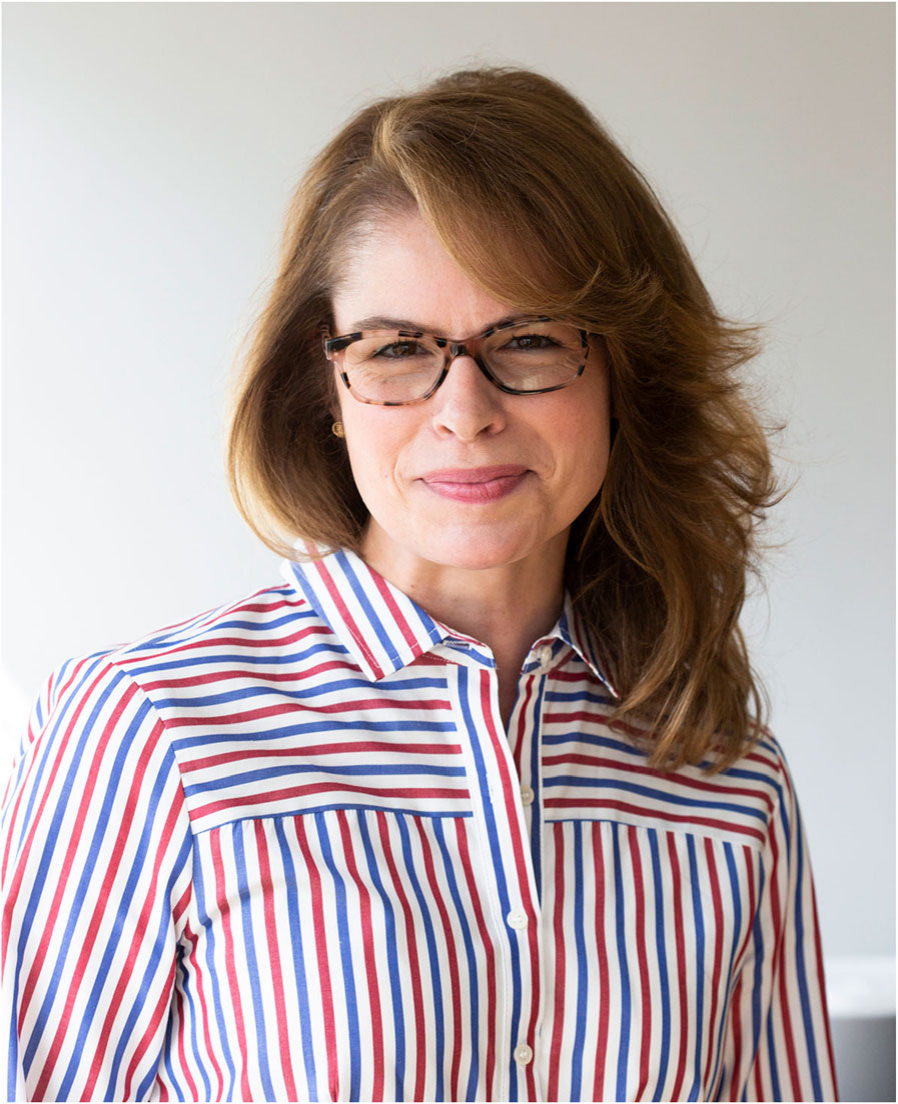
Rachel Reinhardt is the Vice President for Communications at the Ludwig Institute for Cancer Research. Prior to that, she held communications leadership positions at the Juvenile Diabetes Research Foundation, the International AIDS Vaccine Initiative, and France Telecom North America. Rachel is a graduate of Yale University with a dual degree in French and International Studies

We thank all the contributors to this Forum article for their thoughtful and significant input, and for helping to drive a meaningful discussion.

We look forward to catalyzing additional interdisciplinary discussions in cancer prevention. Such dialogue will hopefully help identify opportunities to further invigorate research activities towards understanding the interrelationships of obesity, nutrition, and physical activity in cancer prevention, and how this knowledge might best be utilized to advance public health.

## Data Availability

Not applicable.
